# Modified Mesenchymal stem cell, platelet-rich plasma, and hyaluronic acid intervention in early stage osteoarthritis: A systematic review, meta-analysis, and meta-regression of arthroscopic-guided intra-articular approaches

**DOI:** 10.1371/journal.pone.0295876

**Published:** 2024-03-08

**Authors:** Kevin Christian Tjandra, Robin Novriansyah, I. Nyoman Sebastian Sudiasa, Ardiyana Ar, Nurul Azizah Dian Rahmawati, Ismail Hadisoebroto Dilogo

**Affiliations:** 1 Department of Medicine, Faculty of Medicine, Universitas Diponegoro, Semarang, Indonesia; 2 Kariadi General Hospital, Semarang, Indonesia; 3 Department of Surgery, Faculty of Medicine, Universitas Diopnegoro, Semarang, Indonesia; 4 Stem Cell Medical Technology Integrated Service Unit, Cipto Mangunkusumo Central Hospital, Faculty of Medicine Universitas Indonesia, Jakarta, Indonesia; 5 Stem Cell and Tissue Engineering Research Cluster Indonesian Medical Education and Research Institute (IMERI) Universitas Indonesia, Jakarta, Indonesia; 6 Department of Orthopaedic and Traumatology, Cipto Mangunkusumo General Hospital, Faculty of Medicine Universitas Indonesia, Jakarta, Indonesia; Sheikh Hasina National Institute of Burn & Plastic Surgery, BANGLADESH

## Abstract

**Background:**

Mesenchymal stem cells (MSCs) hold promise for osteoarthritis (OA) treatment, potentially enhanced by combining them with platelet-rich plasma (PRP) and hyaluronic acid (HA). This study aimed to assess the synergy of MSCs, PRP, and varying HA doses, and determine optimal MSC sources to treat early-stage OA in the perspective of Lysholm score, VAS Score, KSS score, and WOMAC score.

**Method:**

Original articles from 2013 to 2023 were screened from four databases, focusing on clinical trials and randomized controlled trials. The Risk of Bias in Non-randomized Studies—of Interventions (ROB-2) tool evaluated bias, and a PICOS criteria table guided result construction. Revman 5.4 analyzed outcomes such as Lysholm score, VAS score, KSS, WOMAC score, cartilage volume, and defect size using MRI. This systematic review adhered to PRISMA guidelines.

**Result:**

Nine studies met the final inclusion criteria. Meta-analysis revealed a significant improvement in Lysholm score (MD: 17.89; 95% CI: 16.01, 19.77; I2 = 0%, P = 0.56), a notable reduction in VAS score (MD: -2.62; 95% CI: -2.83, -2.41; I2 = 99%, P < 0.00001), elevated KSS (MD: 29.59; 95% CI: 27.66, 31.52; I2 = 95%, P < 0.0001), and reduced WOMAC score (MD: -12.38; 95% CI: -13.75, -11.01; I2 = 99%, P < 0.0001).

**Conclusions:**

Arthroscopic guided high-dose subchondral application of primary cultured synovial MSCs in popliteal PRP media with HA effectively regenerates cartilage defects and improves clinical outcomes in early-stage osteoarthritis. Clarification of MSC sources and quantities enhances the understanding of this promising treatment modality.

## Introduction

Osteoarthritis is a chronic inflammatory joint disease due to the degradation of the cartilage that causes joint pain and limitation in movement [1]. Over three decades, the global prevalence of OA increased by 113.25% from 1990–2019 with an estimated 527.81 million people [2]. The pathogenesis of OA is complex due to the multifactorial factors that can influence the occurrence and progression of the disease, including genetic, cellular, biochemical, and immunological. However, the main pathogenesis pathway that worsens the OA condition is the calcification of the cartilage defect. Thus, cartilage repair may prevent the progression of osteoarthritis [3, 4].

The pharmacological conservative therapy does not provide significant benefits and the side effects that can arise also need to be considered in its application [5]. Furthermore, pharmacotherapy itself is not able to solve the main problem of OA, cartilage damage. Surgical techniques such as total knee replacement can be performed but the risk of persistent pain or loss of joint function reaches 20% [6]. Thus, less invasive procedure with promising result is needed to treat OA.

Mesenchymal stem cells (MSCs) as the regenerative agent is one of the solution of knee OA treatment because of its ability to regenerate cartilage defect [7]. Among various MSC sources, synovial MSC is a promising cell source for cartilage repair. Synovial MSCs have superior chondrogenic ability compared to MSCs from other tissues [8]. Synovial tissue has the greatest potential for differentiation into chondrogenic cells and proliferation. The synovium is a thin membrane that covers the inside of the joint and has a high regenerative activity. According to previous studies, the amount of MSCs in synovial fluid is increased in knees with OA that proving its regeneration activity [9]. Transplantation of large numbers of synovial MSCs into injured cartilage tissue can enhance the natural healing process. From previous studies, synovial MSCs were transplanted arthroscopically and led patients to return to daily life and sports activities earlier than patients with invasive surgery [8]. Therefore, This study aimed to assess the synergy of MSCs, PRP, and varying HA doses, and determine optimal MSC sources to treat early-stage OA in the perspective of the Lysholm score, VAS Score, KSS score, and WOMAC score.

## Material and method

### Registration

This systematic review and meta-analysis adhered to the Preferred Reporting Items for Systematic Reviews and Meta-Analyses (PRISMA) guidelines [10]. The study was registered on the Open Science Framework (OSF) on August 8, 2023, with the assigned registration number https://doi.org/10.17605/OSF.IO/HS4RZ.

### Eligibility criteria

Original content from 2013 to 2023 was included, encompassing autologous clinical trials and randomized controlled trials (RCTs). Excluded materials comprised technical reports, editor responses, narrative reviews, systematic reviews, meta-analyses, non-comparative research, in silico studies, in vitro studies, in vivo studies, scientific posters, study protocols, and conference abstracts. Non-English, non-full-text, and unrelated papers not addressing the application of Mesenchymal Stem Cells (MSCs) to treat knee osteoarthritis were also excluded. The selected articles were evaluated based on the PICO criteria: i) patient with osteoarthritis or cartilage damage; ii) intervention of intra-articular intervention or arthroscopic guided MSCs application; iii) comparison, standard care without synovial stem cell application (placebo); and iv) outcomes, treatment evaluation using AMADEUS MRI score, VAS score, Lysholm score, Cartilage volume, size of cartilage defect, Knee Society Clinical Rating System Score (KSS), and WOMAC index.

### Outcome measure

The efficacy evaluation encompassed various outcomes, including AMADEUS MRI score, VAS score, Lysholm score, Cartilage volume, size of cartilage defect, Knee Society Clinical Rating System Score (KSS), and WOMAC index. These measures were selected to comprehensively assess the impact of the therapy on cartilage regeneration and clinical outcomes in osteoarthritis patients.

### Index test

Studies providing data on the application and evaluations of synovial mesenchymal stem cells in osteoarthritis were included.

### Reference standard

The reference standard involved professional research using randomized controlled clinical studies (RCTs) or clinical trials to assess the transition of mesenchymal stem cells in osteoarthritis outcomes.

### Data sources and search

Research was gathered from Pubmed, SpringerLink, Google Scholar, and Science Direct databases. The study not explored EMBASE and Cochrane databases due to potential resource limitations, a focus on well-established databases, and a specific study design and scope. The search covered the period from the establishment of each database until May 22, 2023. Medical Subject Headings (MeSH) keywords were employed, and the keywords used for each database are listed in Table 1.

**Table 1:** Keyword used in literature searching.

Database	
Pubmed	Osteoarthritis and Synovial Membrane and Stem Cell
Springerlink	
Science Direct	
Google Scholar	

### Selection process

Four independent reviewers (KCT, AA, NADR, and INSS) and one validator (RN) collaborated in combining outcomes from four databases, followed by complete text and abstract screening. The inter-rater and self-rater reliability were performed to solve the disagreements during the article selection process. The PRISMA flow chart documented the research selection procedures.

### Data collection process

After the final screening, relevant information from studies was retrieved and entered into a Google Spreadsheet, including author, year, country, study design, sample size, gender, mean age, intervention name, comparison, and outcomes.

### Study risk of bias assessment (qualitative synthesis)

The Cochrane Risk of Bias 2 For Randomized Trials (ROB-2) tool was utilized by five independent reviewers (KCT, RN, AA, NADR, INSS, and IHD) to assess the bias of included studies. Discrepancies were resolved through discussion. Studies with a high risk of bias were excluded.

### Reporting bias assessment

No critical overall bias was identified during ROB-2 assessment, as conducted by KCT, AA, NADR, INSS, and RN. No articles were removed at this stage.

### Quantitative synthesis

Outcomes were presented as mean difference (MD) and standard deviation (SD) with a 95% confidence interval (CI) in the characteristic table. A fixed-effect model (FEM) was employed for homogenous studies (I2 < 40%), and a random-effect model (REM) was used for heterogeneous studies. The pooled mean difference estimate was presented in a forest plot.

### Regression analysis

*Data analysis using regression analysis* is a statistical approach that is commonly used to understand the relationship between one or more independent variables and the dependent variable. The regression model used is linear regression, with the dependent variable "MRI Score for cartilage defect" and independent variables involving "Lysholm Score," "Cartilage Volume (mm^3)," "Size of Cartilage Defect," "WOMAC Score," "Visual Analog Scale," and "Knee Society Clinical Rating System Score (KSS)." The selection of these variables is based on theoretical considerations and relevance to the objectives of the analysis. Data regression analysis using STATA 17.

## Result

### Study selection

**Fig 1:**
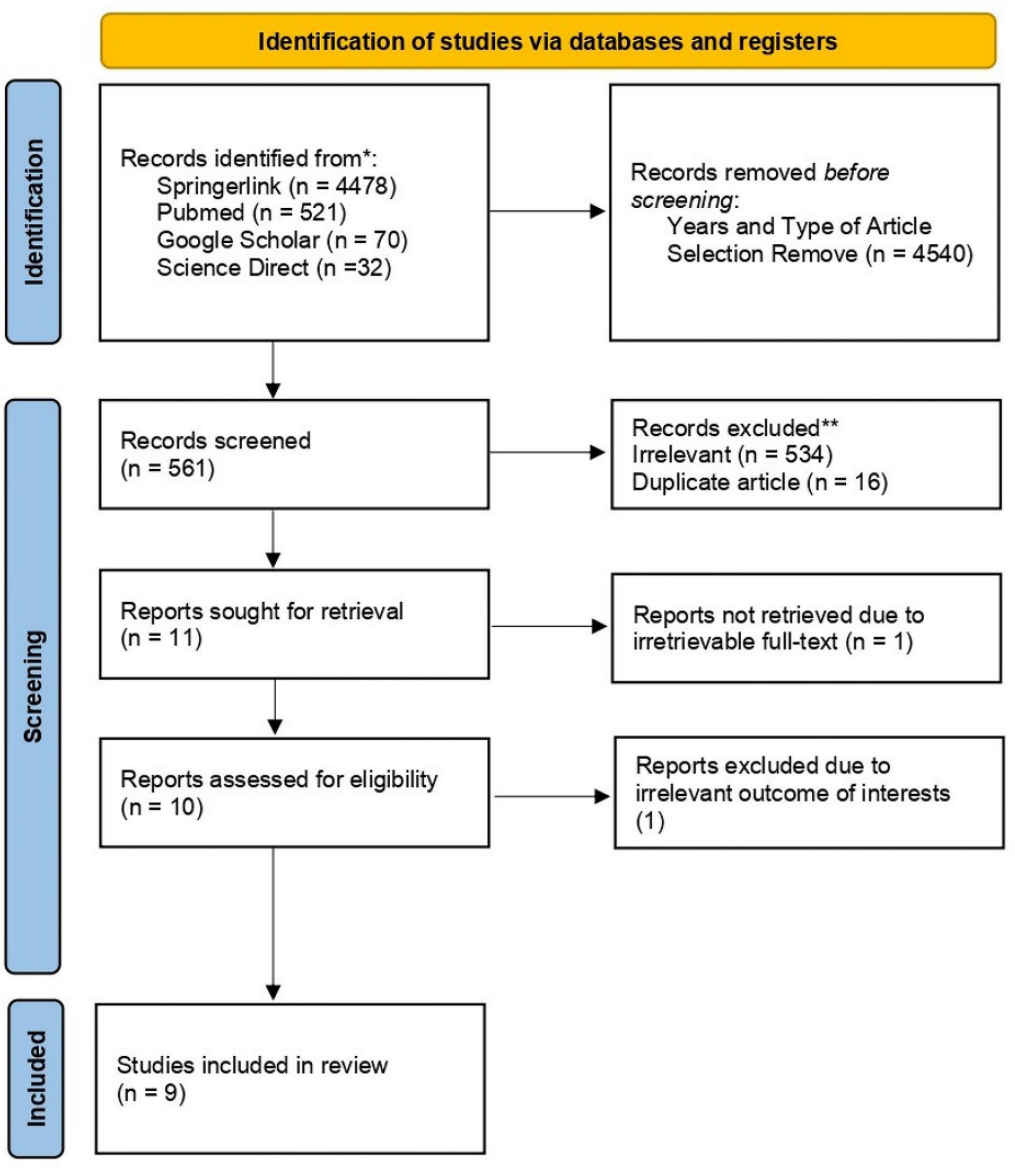
PRISMA 2020 flow diagram.

A comprehensive literature search across Springerlink, Pubmed, Google Scholar, and ScienceDirect initially yielded 5,101 papers. Automated filtering methods excluded 4,540 non-clinical trials and non-randomized controlled trial research. After a meticulous review of titles, abstracts, and full texts, 534 irrelevant subjects and 16 duplicate articles were excluded. Additionally, one unretrieved item due to no related subscription of our institution available and one irrelevant outcome (not available of Lysholm score, VAS score, KSS, and WOMAC Score, AMADEUS MRI Score, Cartilage Volume, Cartilage defect) research paper were removed, leading to the inclusion of nine studies in this systematic review and meta-analysis. The PRISMA flow diagram in Fig 1 illustrates the research selection process and the reason of excluded articles.

### Study characteristics

The systematic review comprised nine studies with a total of 315 participants conducted across different countries, including Japan, South Korea, France, China, USA, Iran, Singapore, and Spain. A detailed breakdown of the study characteristics, including the PICO elements, is provided in Table 2.

**Table 2:** Studies characteristic.

No.	Title	Author; Year	Country/Place	Study Design	Study Period	Population				
						Sample size (n)	Number of Knee	Male (%)	Mean Age (years) (Mean±SD)	Disease Stage (intervention)
1	Arthroscopic Transplantation of Synovial Stem Cells Improves Clinical Outcomes in Knees With Cartilage Defects	Sekiya I, et al.; 2015	Japan	Clinical Trial	April 2008-April 2011	10	10	50.00%	Intervention and Control: 34.1±9.5	ICRS Grade 3 and 4 (10)
2	Intra-Articular Injection of Mesenchymal Stem Cells for the Treatment of Osteoarthritis of the Knee: A Proof-of-Concept Clinical Trial	Hyunchul C, et al.; 2014	South Kprea	Clinical Trial	March 2009-September 2011	18	18	16.67%	Intervention: Low-Dose (63±8.6) Mid-Dose (65±6.6) High-Dose (61±6.2)	KL Grade 3 (12); KL Grade 4 (6)
3	Subchondral bone or intra-articular injection of bone marrow concentrate mesenchymal stem cells in bilateral knee osteoarthritis: what better postpone knee arthroplasty at fifteen years? A randomized study	Hernigou P, et al.; 2020	France	Randomized Clinical Trial	2000–2005	60	120	41.67%	Intervention and Control: 61	KL Grade 1 (22); KL Grade 2 (40); KLGrade 3 (38); KL Grade 4 (20)
4	Intra-articular platelet-rich plasma combined with hyaluronic acid injection for knee osteoarthritis is superior to PRP or HA alone in inhibiting inflammation and improving pain and function	Xu Z, et al.; 2020	China	Clinical Trial	June 2016-June 2017	78	122	41.82%	Not Stated	IIIC (19); IV (12)
5	Clinical Efficacy of intra-articular Mesenchymal Stromal Cells for the Treatment of Knee Osteoarthritis	Garza JR, et al.; 2020	USA	Randomized Clinical Trial	December 2002-March 2004	39	39	43.59%	Intervention and Control: 59.0±9.9	KL Grade 2 (12); KL Grade 3 (27)
6	Intra-articular injection of Autologous Mesenchymal Stem Cells in Six Patients with Knee Osteoarthritis	Emededin M, et al.; 2012	Iran	Clinical Trial	Not stated	6	6	0,00%	54.56	Not Stated
7	Intra-articular injection of Mesenchymal Stem Cells for the Treatment of Osteoarthritis of the Knee	Hyunchul C, et al.; 2017	South Korea	Prospective Cohort, Randomized Clinical Trial	Not stated	18	18	16.67%	61.8±6.6	Not Stated
8	Injectable Cultured Bone Marrow Derived Mesenchymal Stem Cells in Varus Knees With Cartilage defects Undergoing High Tibial Osteotomy: A Prospective, Randomized Controlled Clinical Trial With 2 Years’ Follow-up	Wong KL, et al.; 2013	Singapore	Prospective Cohort, Randomized Clinical Trial	Not stated	56	56	48.21%	Not Stated	ICRS grade 2 (8); ICRS grade 3 (16); ICRS grade 4 (32)
9	Intra-articular injection of two different doses of autologous bone marrow mesenchymal stem cells versus hyaluronic acid in the treatment of knee osteoarthritis: multicenter randomized controlled clinical trial (phase IIII)	Espinosa JML, et al.; 2016	Spain	Randomized Clinical Trial	August 2012-October 2014	30	30	63.33%	Control: 60.3 Low-dose: 65.9 High-dose: 57.8	Not stated

### Risk of bias in studies

**Fig 2:**
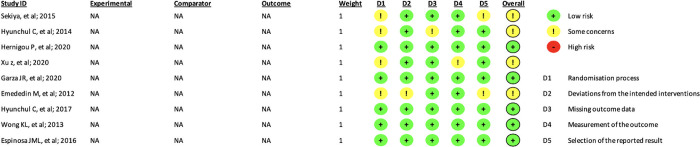
Risk of bias assessment.

Each clinical trial and randomized controlled trial underwent a thorough assessment using the ROB-2 method. Three studies were identified with some concern of bias due to an unclear randomization process. The risk-of-bias assessment is visually represented in Fig 2.

### Study result summaries

The nine studies included in this systematic review are by Sekiya et al. (2015), Hyunchul C et al. (2014), Hernigou et al. (2020), Xu Z et al. (2020), Garza JR et al. (2020), Emededin M et al. (2012), Hyunchul C et al. (2017), Wong KL et al. (2013), and Espinosa JML, et al. (2016). A brief overview of each study is provided in Table 2.

### Lysholm score

**Fig 3:**
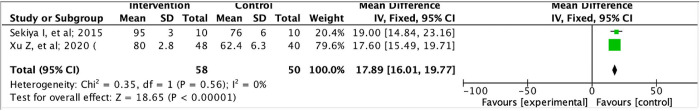
Forest plot of Lysholm score between intervention vs control group.

A meta-analysis of two studies assessing the efficacy of synovial fluid stem cells in relieving disability symptoms showed a significant effect (P < 0.00001) with a pooled mean difference (MD) of (17.89) (95% CI: 16.01, 19.77). Heterogeneity was found (I2 = 0%; P < 0.00001) (Fig 3).

### Visual analog scale (VAS)

**Fig 4:**
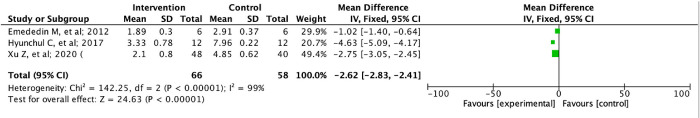
Forest plot of VAS score between intervention vs control group.

A meta-analysis of three studies analyzing the efficacy of synovial fluid stem cells in reducing clinical pain in OA patients demonstrated a significant effect (P < 0.00001) with a pooled MD of -2.62 (95% CI: -2.83, -2.41). Heterogeneity was found (I2 = 0%; P < 0.00001) (Fig 4).

### Knee society clinical rating system score (KSS)

**Fig 5:**
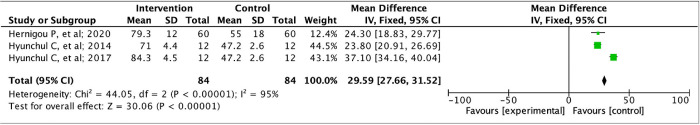
Forest plot of KSS score between intervention and control group.

The forest plot using KSS showed a significant effect (P < 0.00001) with a pooled MD of 29.59 (95% CI: 27.66, 31.52). Heterogeneity was found (I2 = 95%; P < 0.00001) (Fig 5).

### WOMAC score

**Fig 6:**
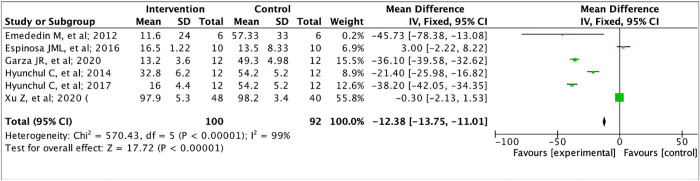
Forest plot of WOMAC score between intervention and control group.

The forest plot analyzing WOMAC score showed a significant effect (P < 0.00001) with a pooled MD of -12.38 (95% CI: -13.75, -11.01). Heterogeneity was found (I2 = 99%; P < 0.00001) (Fig 6).

### Regression analysis

#### 1. Validity and Reliability Test

a. Validity Test

**Fig 7:**
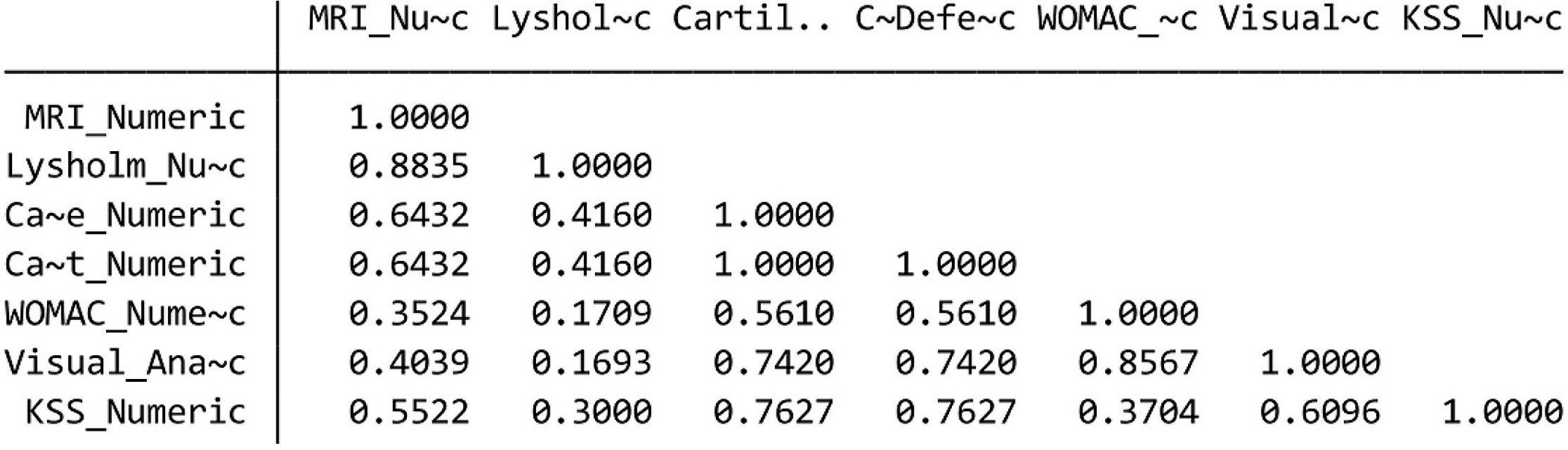
Pearson correlation.

Pearson correlation measures the degree to which two variables relate to each other in a linear relationship. If the Pearson correlation coefficient is close to 1 (positive), this indicates a strong positive relationship between two variables, meaning that they tend to move together in the same direction. The validity value of the test shows that there is a positive relationship between all variables (Fig 7).

b. Reliability Test

**Fig 8:**
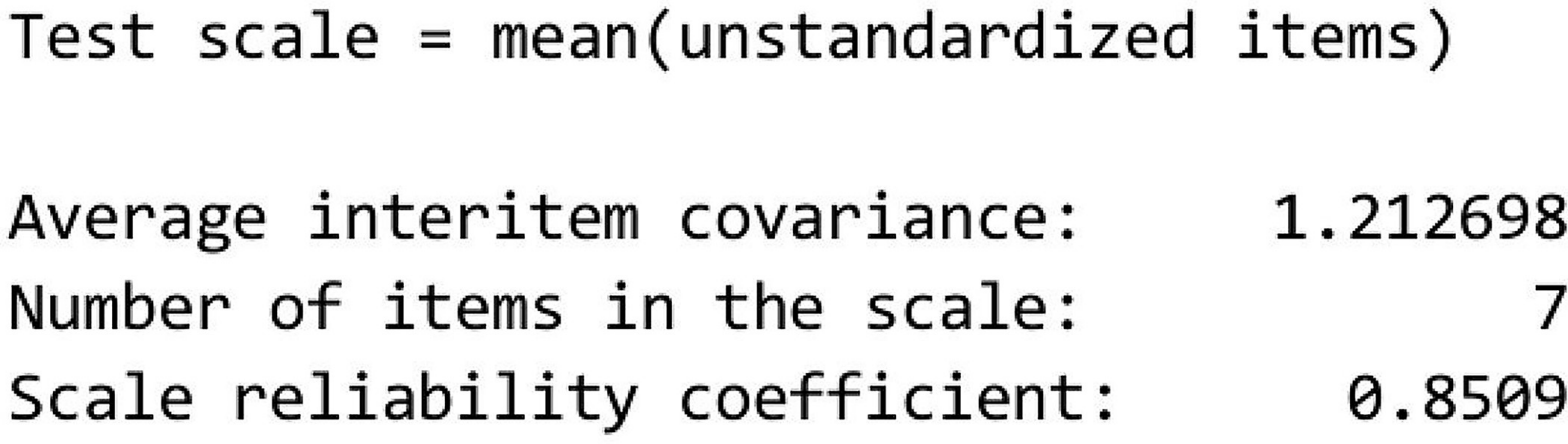
Alpha Cronbach.

Reliability is a measure of stability and consistency, which are the dimensions of a variable. Cronbach’s alpha coefficient measures the extent to which the variables used in a measurement tool are consistent or reliable. The alpha value ranges from 0 to 1, and the closer it is to 1, the higher the reliability of the measurement tool. High alpha values are usually considered good, and numbers above 0.70 are considered reliable. In this case, 0.8509 is Cronbach’s alpha value, which shows the reliability between these variables. The alpha value ranges between 0 and 1, and the closer it is to 1, the higher the reliability (Fig 8).

#### 2. Linear Regression Analysis

**Fig 9:**
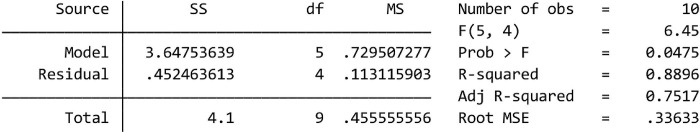
Linear regression results.

The linear regression test is a test of data that consists of independent variables and dependent variables, where these variables are causal (influential) (Fig 9).

The linear regression equation in this research is:

MRIScore=β0+β1×LysholmScore+β2×CartilageVolume+β3×SizeofCartilageDefect+β4×WOMACScore+β5×VisualAnalogScale+β6×KSSScore+ε

In this equation:

- MRI Score is the dependent variable to be explained.
- β0 is the intercept, namely the value of the MRI Score, when all independent variables are equal to 0.
- β1,β2,…,β6 are regression coefficients that measure the average change in MRI ScoreMRI Score for every one unit change in the corresponding independent variable, assuming all other variables remain constant.
- Lysholm Score, Cartilage Volume, Size of Cartilage Defect, WOMAC Score, Visual Analog Scale, KSS ScoreLysholm Score, Cartilage Volume, Size of Cartilage Defect, WOMAC Score, Visual Analog Scale, KSS Score are independent variables.
- ε is a random error.

Based on the results above, the regression equation created is:

MRIScore=0.229+0.338LysholmScore+0.102CartilageVolume+0.130SizeofCartilageDefect+0.028WOMACScore–0.018VisualAnalogScale+0.090KSSScore+ε

#### 3. Classic Assumption Test

**a. Multicollinearity Test**

**Fig 10:**
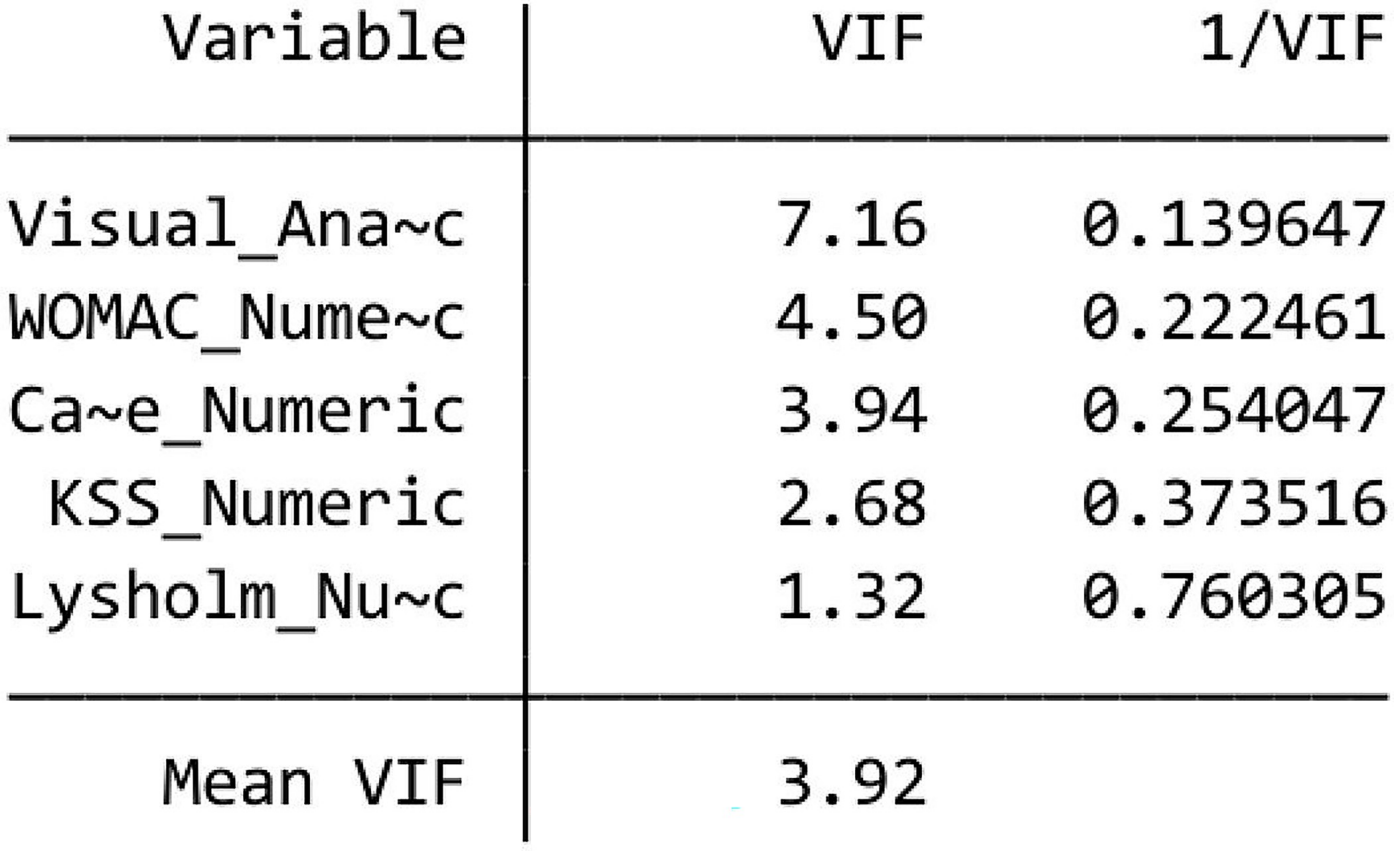
VIF.

The multicollinearity test is used to determine whether there is a high correlation between two or more independent variables in the regression model. One way to carry out a multicollinearity test is to use the variance inflation factor (VIF) (Fig 10). A high VIF (above 10) can indicate multicollinearity.

Based on the results of data analysis, the VIF value < 10 means there are no symptoms of multicollinearity in the model.

b. Heteroscedasticity Test

**Fig 11:**
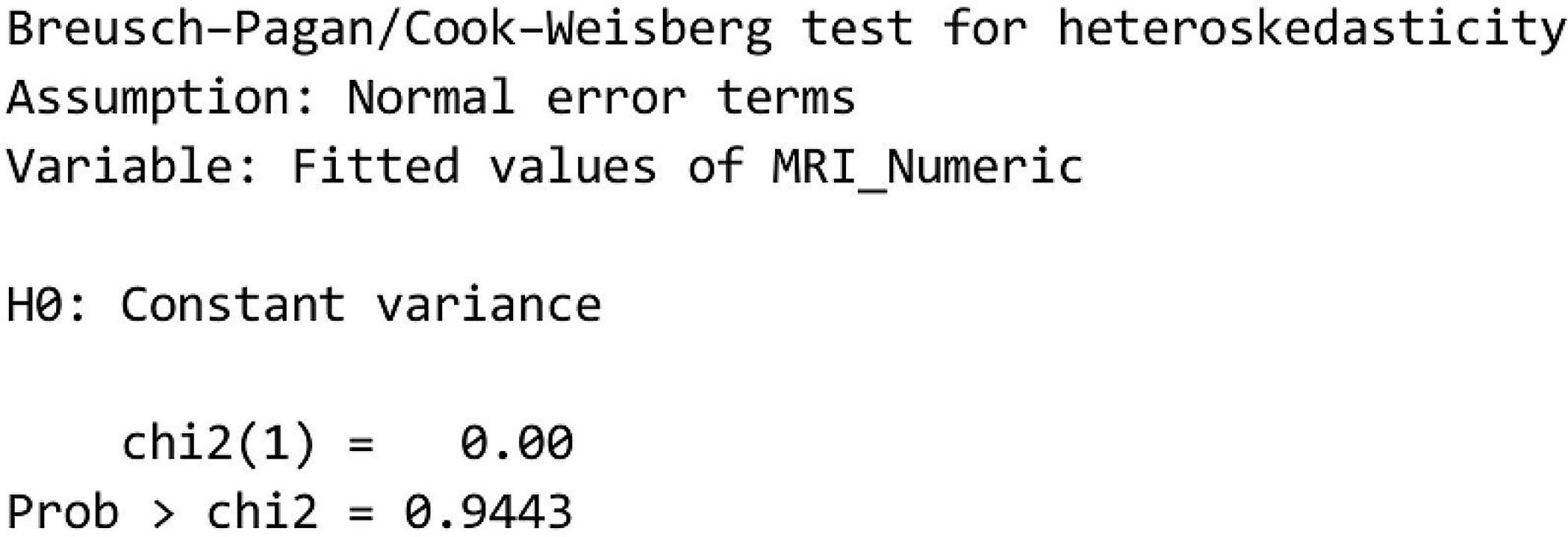
Breusch-pagan test.

The heteroscedasticity test is used to identify whether there is heterogeneous variation in the residual error of the regression model. One way to carry out a heteroscedasticity test is to use the Breusch-Pagan test (Fig 11).

Based on the results of data analysis, the value of Prob > chi2 = 0.9443 means that there is no heteroscedasticity in the model.

#### 4. Hypothesis test

Hypothesis testing in this research is to determine whether the hypothesis is accepted or rejected. Hypothesis testing is carried out through:

a. F Test

The F test in linear regression analysis aims to determine the effect of the independent variable on the dependent variable simultaneously, which is shown in the ANOVA table.

**Fig 12:**
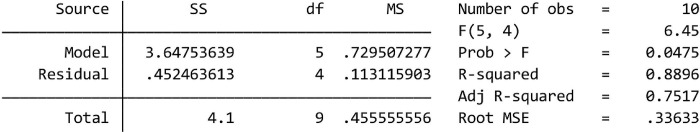
F test.

If the significance value is <0.05 or 0.0475, then the decision is that the hypothesis is accepted or the independent variable simultaneously has a significant effect on the dependent variable as seen in Fig 12.

b. T-test

**Fig 13:**
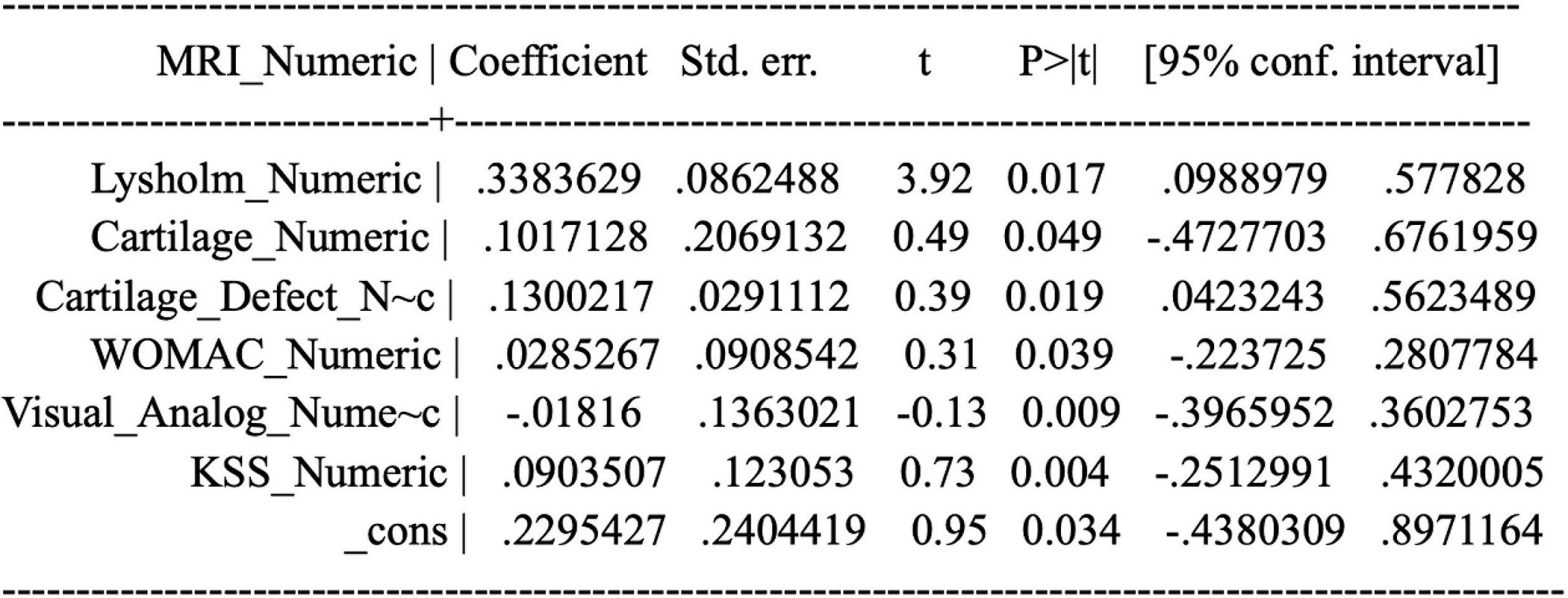
T-test.

The t-test is used to determine the influence of each independent variable partially on the dependent variable, as shown by the coefficients table. The significance value of the t-test for each variable is <0.05. Then, the decision is that the hypothesis is accepted or all independent variables partially have a significant effect on the dependent variable on Fig 13.

c. Coefficient of Determination (R2)

**Fig 14:**
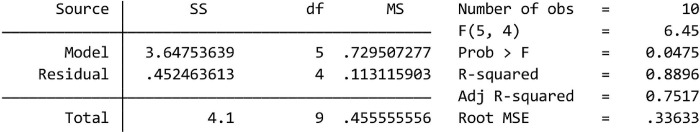
Coefficient of determination (R2).

These results show an R-squared value = 0.8896, which means that all independent variables can explain the dependent variable by 88.96%. So, the remaining 100%-88.96% = 11.04% is influenced by other variables outside the regression model on Fig 14.

Based on this analysis, it can be concluded that simultaneously and partially, the independent variables, namely Lysholm Score, Cartilage Volume, Size of Cartilage Defect, WOMAC Score, Visual Analog Scale, and Knee Society Clinical Rating System Score, have a significant effect on the dependent variable, namely MRI Score for cartilage defects. In other words, the combination of these independent variables together or individually makes a significant contribution to the variation in the MRI Score for cartilage defects.

## Discussion

### Synovial mesenchymal stem cell

Synovial Mesenchymal Stem Cells (Sy-MSCs) originate from the mesenchyme, which is an embryonic connective tissue that gives rise to various types of cells, including bone, cartilage, and adipose tissue. Synovial MSCs have the ability to self-renew and differentiate into several types of cells from the mesenchymal lineage, including osteoblasts (bone cells), synovial (cartilage cells), and adipocytes (fat cells) [11]. They also exhibit immunomodulatory properties, meaning they can regulate the immune response. Synovial MSCs can be isolated from synovial fluid or the synovial membrane/tissue of joints such as the knee, hip, or shoulder. Various methods, including aspiration or biopsy, can be used to obtain these cells [12].

Synovial MSCs have garnered significant interest in regenerative medicine and tissue engineering due to their ability to differentiate into different cell types [13]. It has been shown that Sy-MSCs had a greater proliferation capacity and stronger chondrogenic potential than Bone Marrow-MSCs and Adipose Tissue-MSCs, as well as less hypertrophic differentiation than bone Bone Marrow-MSCs. In addition, pellet cultures were significantly larger from Sy-MSC than Bone Marrow-MSCs in patient-matched comparisons. They hold promise for the repair and regeneration of damaged or diseased joint tissues, such as cartilage and bone. They are being explored as potential treatments for conditions such as osteoarthritis, rheumatoid arthritis, and joint injuries [14].

Synovial MSCs play an important role in the pathophysiology and treatment of osteoarthritis (OA). In osteoarthritis, the synovial membrane undergoes changes, including inflammation and hyperplasia [15]. Synovial MSCs participate in the repair and maintenance of joint tissues, including articular cartilage. However, in OA, the regenerative capacity of synovial MSCs is often impaired due to factors such as aging, inflammation, and abnormal joint microenvironment. Synovial MSCs have chondrogenic potential, meaning they can differentiate into chondrocyte-like cells responsible for producing and maintaining the cartilage matrix [16–18].

Synovial MSCs hold promise for the treatment of OA. They can be isolated from synovial fluid or the synovial membrane through minimally invasive procedures [19]. These cells can be expanded in the laboratory and then transplanted back into the joint to promote tissue repair and regeneration.

Synovial Mesenchymal Stem Cells (Sy-MSCs) offer a promising avenue in regenerative medicine due to their origin from embryonic connective tissue and their ability to differentiate into various cell types. Synovial MSCs, derived from synovial fluid or membrane, possess superior chondrogenic potential compared to other MSC sources, making them an ideal candidate for cartilage repair. These cells, crucial for joint tissue maintenance, exhibit immunomodulatory properties, contributing to their role in the pathophysiology and treatment of osteoarthritis (OA). However, challenges such as impaired regenerative capacity in OA conditions necessitate further exploration of these cells’ therapeutic potential [20].

### Application of synovial stem cell in treating osteoarthritis

Osteoarthritis may develop as a result of articular cartilage injuries, which are a common clinical issue. Despite the variety of surgical intervention techniques, each has disadvantages of its own: Bone marrow stimulation results in poor structural quality of the repaired cartilage, donor site morbidity in mosaicplasty, and loss of the chondrogenic phenotype of expanded chondrocytes in autologous chondrocyte implantation [21]. One potential method for enhancing cartilage injury repair is stem cell therapy. Mesenchymal stem cells (MSCs), which can be isolated from various mesenchymal tissues, especially synovial membranes are one of the potential therapeutic cells.

The potential of synovial MSCs can be maximized by combining with other intraarticular regeneration agents, including, Platelet-Rich Plasma and hyaluronic acid [22]. This combination might be a great solution to treat cartilage damage in Osteoarthritis and prevent the overuse of hyaluronic acid that fastens the OA disease progression. A small amount of hyaluronic acid intraarticular application is able to stimulate cartilage regeneration. However, this small amount of HA is not enough to stop the disease progression, but the more hyaluronic acid is used the more VEGF production occurs. The high amount of VEGF will calcify the cartilage and convert it into osteocytes which worsen osteoarthritis [23].

Among all 4 Kellgern Lawrence classifications, this non-operative treatment is useful in OA grades 1–3. The earlier stage the greater outcome will be obtained [24]. It is because of this treatment strategy, that Modified Synovial Mesenchymal Stem Cells act as a cartilage regenerative agent that prevents further OA progression [8].

Osteoarthritis often results from articular cartilage injuries, prompting the exploration of stem cell therapy, particularly using Mesenchymal Stem Cells (MSCs) isolated from synovial membranes. The study proposes a combination of synovial MSCs with intraarticular regeneration agents, including Platelet-Rich Plasma (PRP) and hyaluronic acid (HA). This synergistic approach aims to enhance cartilage injury repair, prevent OA progression, and optimize the therapeutic potential of synovial MSCs.

### Effication of MSCs in treating osteoarthritis

In recent years, the potential use of mesenchymal stem cells as a therapeutic approach for OA has gained significant attention. Compared to conventional bone marrow MSCs, Synovial mesenchymal stem cell (SFCs) has more efficacy as proven in the study conducted by Sekiya, et al. (2015) [8]. It’s because synovial fluid cells have more similarity in synovial environment than other sources of cells. Thus, it can exhibit multipotency where each cell can differentiate into various cell types including chondrocytes, which are crucial for the maintenance and repair of cartilage. The regenerative properties of SFSCs are important in a joint inflammation disease such as osteoarthritis. A study by Kim et al. (2019) demonstrated that SFSCs derived from OA patients could differentiate into chondrogenic lineage cells and promote cartilage matrix formation [24]. Moreover, another study by Qiu et al. (2021) reported that SFSCs combined with hyaluronic acid injections resulted in improved cartilage regeneration and enhanced functional outcomes in a rabbit model of OA [25]. Then, the efficacy of this treatment can be maximized by the application of appropriate high-dose MSCs (1 x 10^8^ cells) with subchondral technique compared to intra-articular injection as proven by a study conducted by Hernigou P, et al. (2020) [26].

However, studies exploring the efficacy of SFSCs in human trials are still relatively limited. A phase I/II clinical trial conducted by Jo et al. (2019) involved intra-articular injection of SFSCs in patients with knee OA [27].

This study reported significant improvements in pain, function, and cartilage quality, demonstrating the potential of MSCs as a new treatment modality for OA. Studies conducted by Sekiya et al. (2015) and Xu, et al. (2020) has proven elevation of the Lysholm score va which assesses disability in OA patients proved to be statistically significant (P <0.001) increased by polled MD (17.89) [95% CI:16.01, 19.77] compared to the control group [8, 28].

In addition, MSCs were also proven to be significantly effective in reducing VAS scores as proven by studies conducted by Emededin M, et al. (2012), Hyunchul, et al. (2017), and Xu Z, et al. (2020) showed the statistical effectiveness of Intra-articular Injection of Autologous MSCs (P <0.00001) with pooled MD (-2.62) (95% CI: -2.83, -2.41) [28–30]. Proven by MRI images, post-injection cartilage thickness increased in three of the six patients. Xu et al. (2020) showed that the efficacy of intra-articular platelet-rich plasma (PRP) SFSC would increase when combined with hyaluronic acid and (PRP) as demonstrated by a significant reduction in VAS scores in the PRP+HA group at 24 months afterward. injection, PRP+HA was more effective than HA and PRP alone in relieving pain (P = 0.0001) [28]. In addition, significant improvements were also observed in the PRP+HA group after 1, 6, 12, and 24 months (P < 0.0001).

MSCs can also improve the WOMAC score which assesses the severity of clinical problems in osteoarthritis patients. The results of the study by Emededin, et al. (2012), Espinosa, et al. (2016), Garza, et al. (2020), Hyunchul, et al. (2014), Hyunchul, et al. (2017), and Xu, et al. (2020) showed a clinically meaningful reduction of WOMAC score as much pooled MD (-12.38) (95% CI: -13.75, -11.01) [29–33]. In addition, KSS function score evaluation from Hernigou, et al. (2020), Hyunchul, et al. (2014), and Hyunchul, et al. (2017) increased significantly from as much pooled MD (29.59) (95% CI: 27.66, 31.52) [26, 30, 33].

The discussion underscores the potential of synovial MSCs in treating osteoarthritis, emphasizing their superiority over conventional bone marrow MSCs. Notably, the multipotency of synovial fluid cells, particularly their ability to differentiate into chondrocytes, is crucial for cartilage maintenance and repair. Studies presented in the discussion highlight the positive outcomes of intra-articular injection of synovial MSCs, including improved Lysholm scores, reduced Visual Analog Scale (VAS) scores, enhanced Knee Society Clinical Rating System (KSS) scores, and relief of symptoms measured by WOMAC scores [34].

The discussion also references studies demonstrating the effectiveness of combining synovial MSCs with other agents such as PRP and hyaluronic acid. This combination approach aims to maximize the therapeutic impact on cartilage damage in osteoarthritis. The study emphasizes the importance of appropriate dosages and techniques, such as subchondral injection, to maximize the efficacy of MSCs.

## Strength and limitation

This study is the first systematic review and meta-analysis that assessed the efficacy of synovial fluid stem cells in treating osteoarthritis. This study also included studies from various countries showing this therapy’s universal applicability. Although this study also has several limitations such as the limited number of included studies.

## Conclusion

In conclusion, our study highlights the promising potential of arthroscopic guided injection of primary cultured synovial mesenchymal stem cells (Sy-MSCs) in popliteal platelet-rich plasma (PRP) media combined with hyaluronic acid (HA) for the effective regeneration of cartilage defects in the early stages of osteoarthritis. Its efficacy was proven by better outcomes, indicated by the increased score of KSS, reduced number of VAS score, WOMAC score, as well as Lysholm score. While our results present a significant step forward in understanding the therapeutic potential of Sy-MSCs in osteoarthritis treatment, we acknowledge the need for further research and exploration. Questions regarding optimal dosages, long-term efficacy, and broader applicability across different stages of osteoarthritis remain open and should be addressed in future investigations.

## Recommendation

More studies need to be conducted to further evaluate the duration and efficacy of the synovial mesenchymal stem cell as a novel therapy for osteoarthritis.

## Supporting information

S1 Checklist PRISMA 2020 checklist. (DOCX)
